# Simultaneous Improvement and Genetic Dissection of Drought Tolerance Using Selected Breeding Populations of Rice

**DOI:** 10.3389/fpls.2018.00320

**Published:** 2018-03-15

**Authors:** Yanru Cui, Wenying Zhang, Xiuyun Lin, Shizhong Xu, Jianlong Xu, Zhikang Li

**Affiliations:** ^1^Institute of Crop Sciences, National Key Facility for Crop Gene Resources and Genetic Improvement, Chinese Academy of Agricultural Sciences, Beijing, China; ^2^Ningxia Academy of Agriculture and Forestry Sciences, Yinchuan, China; ^3^Rice Research Institute, Jilin Academy of Agricultural Sciences, Changchun, China; ^4^Department of Botany and Plant Sciences, University of California, Riverside, CA, United States; ^5^Shenzhen Institute of Breeding and Innovation, Chinese Academy of Agricultural Sciences, Shenzhen, China

**Keywords:** QTL, drought tolerance, functional genetic units, non-random associations, hidden genetic diversity

## Abstract

Drought is the most important factor limiting rice yield in most rainfed areas of Asia and Africa. Four large BC_2_F_2_ populations consisted of 3,200 individuals, which were derived from crosses between an elite *Geng* variety, Jigeng88, and four donors from three different countries, were screened and progeny tested under severe drought stress, resulting in the development of 72 introgression lines (ILs) with significantly improved yield compared to the recurrent parent Jigeng88. These DT ILs plus four random populations (without drought selection population) from the same crosses were evaluated in replicated trials under both drought stress and non-stress conditions in two environments, and characterized with simple sequence repeat (SSR) markers to understand how directional selection was operating on the genetic variation of DT of rice. Thirteen DT QTLs of large effect were identified based on the significant allelic and genotypic frequency shits in the DT ILs by using the joint segregation distortion method. The 13 QTLs were validated by the genotypic differences at individual QTL in the random populations. Putative genetic networks consisting of 30 loci in 29 functional genetic units underlying DT were detected by *X*^2^ tests and non-random associations between or among DT loci in DT ILs from the four populations. Most large-effect DT QTLs were previously reported and located in the upstream of the genetic networks as putative regulators, and were either mapped to important regulatory genes for DT or drought responsiveness reported previously. In our study, five promising ILs with significantly improved yield were selected under both drought and normal irrigated conditions. The QTLs and their genetic networks underlying DT detected provided useful genetic information for further improving DT and yield using designed QTL pyramiding.

## Introduction

Rice (*Oryza sativa* L.) is the major food crop for more than 700 million people and more than 90% of rice in the world is grown and consumed in Asia (Ji et al., 2012; Palanog et al., 2014). However, with the deterioration of environment, area with severe water shortage is expected to increase. In Asia, about 50% of the rice land is rainfed rice area where the water supply is unpredictable and droughts are common. Rice is sensitive to drought stress at the reproductive stage, when a slight drought stress can cause drastic yield loss (Kamoshita et al., 2008; Palanog et al., 2014). Drought has been a major abiotic stress factor for limiting rice production in rainfed ecosystem. Developing drought tolerance (DT) rice cultivars is the direct and effective way to reduce crop loss.

Developing DT rice varieties is often challenging because of the complexity of DT. In nature, drought stress may occur at any stage of rice growth, and the effect of drought stress on rice are various at different growth stage. The rice species has 12 chromosomes with the whole genome size of 430 Mb. In rice breeding, direct selection for improving DT was often ineffective because rice DT often show a considerable degree of genotype by environment interaction (Fukai and Cooper, 1995). As a result, tremendous efforts have been devoted to genetically dissect DT related secondary traits such as root architecture, leaf water potential and relative water content, etc. Unfortunately, indirect selection for secondary traits have not been effective to improve DT in rice due to poor correlation between the secondary traits and grain yield under drought stress (Palanog et al., 2014). Recent studies showed that direct selection for grain yield (GY) in artificial or natural drought stress conditions is the most effective way for developing DT rice with high yield potential under non-tress conditions (Venuprasad et al., 2007, 2008; Guan et al., 2010). However, it often takes long time for cultivar development using traditional methods of rice breeding on the basis of simple cross making and phenotypic selection. An alternative method of improving breeding effectiveness is to identify quantitative trait loci (QTLs) with large and consistent effects in different populations under drought stress conditions. The identified QTL can be used for marker assistant breeding (MAB) (Venuprasad et al., 2011). Thus, many efforts have been made to identify large-effect QTL affecting DT and develop marker assisted selection systems for improving rice DT (Xu et al., 2005; Yue et al., 2005; Palanog et al., 2014). Again, despite larger numbers of QTL related to DT have been detected, relatively few of them have large and consistent effects for an efficient MAS program.

In order to fill the gap between basic genetic/molecular dissection of DT and improving DT in breeding, a new strategy has been proposed for simultaneous improvement and genetic dissection of complex traits using backcross breeding and marker-facilitated tracking of gene flow from donors to recipients from selection (Li et al., 2005b). In other words, selected breeding progeny will be used to conduct QTL mapping, which have three major advantages over the classical QTL mapping. The first one is the small size of selected breeding populations and thus requires low costs in both genotyping and phenotyping. Second, selected population often has greatly increased power in detecting QTL for the target traits under selection, but much reduced power in detecting QTL for non-target traits. The third and most important one is that mapping QTL using selected population is part of breeding and lines from selected populations are expected to carry beneficial alleles of QTL. Thus, selected lines can potentially become new varieties, but more likely can be used directly as parents in making crosses of “designed QTL pyramiding,” an important step toward breeding by design (Ali et al., 2017).

In this study, we demonstrated again the strategy for simultaneous improvement and genetic dissection of DT of rice in the process of breeding. We reported the development of superior lines with significantly improved grain yield under both drought stress and irrigated conditions as well as providing useful breeding lines and genetic information for further improving rice yield and DT using designed QTL pyramiding.

## Materials and methods

### Development of the plant materials

A superior high yield *Geng* (*japonica*) variety, Jigeng88, which is an elite cultivar commercially grown in Jilin province of China, was used as the recurrent parent (RP) and four varieties collected from China, Malaysia, and IRRI as donors. These donors contained three *Xian* (*indicas*) and one *Geng* variety (Table 1). In the summer of 2005, Jigeng88 (JG88) was crossed with all donors to produce F_1_s on the experimental farm of Ningxia Academy of Agriculture and Forestry Sciences (NAAFS) in Yinchuan (38.5° N, 106.2° E). The F_1_ plants were backcrossed with the RP to produce BC_1_F_1_ population in Sanya (18.3° N, 109.3° E), Hainan Province of China during the winter season of 2005–2006. In the summer of 2006, 25–30 randomly selected plants from each of the BC_1_F_1_ populations were backcrossed with the RP to produce 25–30 BC_2_F_1_ lines. From each of the crosses, 25 BC_2_F_1_ lines were planted (36 plants of each line in a single row) in 2007. Selfed seeds from individual plants of 25 BC_2_F_1_ lines of each cross were bulk harvested to produce a single bulk BC_2_F_2_ population.

**Table 1:** The information of 4 rice backcross populations used for improving drought tolerance in this study.

			**DT selected ILs**						**Random ILs**		
**Donor (code)**	**Subspecies^a^**	**Origin**	**N_1_**^b^	**N_2_**	**SI (%)**^c^	**N_3_**	**DG%**^d^	**H**^e^	**N_4_**	**DG%**	**H**
							**Mean** ± ***SD***			**Mean** ± ***SD***	
IR66897B (I)	*Xian(X)*	IRRI	800	28	3.5	17	13.5 ± 16.8	2.1 ± 3.3	60	10.4 ± 9.8	2.8 ± 3.3
MR77 (II)	*Xian(X)*	Malaysia	800	40	5.0	21	10.2 ± 8.1	1.4 ± 3.0	55	5.9 ± 6.5	1.0 ± 2.6
MR167 (III)	*Xian(X)*	Malaysia	800	29	3.6	10	8.9 ± 16.8	0.0	60	6.4 ± 7.2	0.6 ± 1.5
SN265 (IV)	*Geng(G)*	China	800	38	4.8	24	19.9 ± 11.5	3.6 ± 4.8	60	13.9 ± 8.1	0.9 ± 1.3
Mean				33.8	4.2	19.8	11.5	1.8	58.8	9.2	1.3

a *Subspecies X = Xian (indica) and G = Geng (japonica)*.

b *N_1_is the original size of the BC_2_F_2_ population used for screening drought tolerance. N_2_ is the number of surviving plants initially selected from each population after drought stress treatment at the reproductive stage. N_3_ is the number of selected BC progeny with significantly improved DT as confirmed by progeny testing of their derived BC_2_F_2:3_ ILs under drought stress at the reproductive stage. N_4_ is the size of each of the BC_2_F_2_ random populations used for validation*.

c *SI is the initial selection intensity (proportion of plants selected) in the BC_2_F_2_ populations*.

d *Percentage of the donor genome of the BC_2_F_2_ ILs was calculated based all polymorphic SSR markers used in genotyping*.

e *H, heterozygosity*.

### The screening of the BC_2_F_2_ bulk populations for DT at the reproductive stage

**Figure 1:**
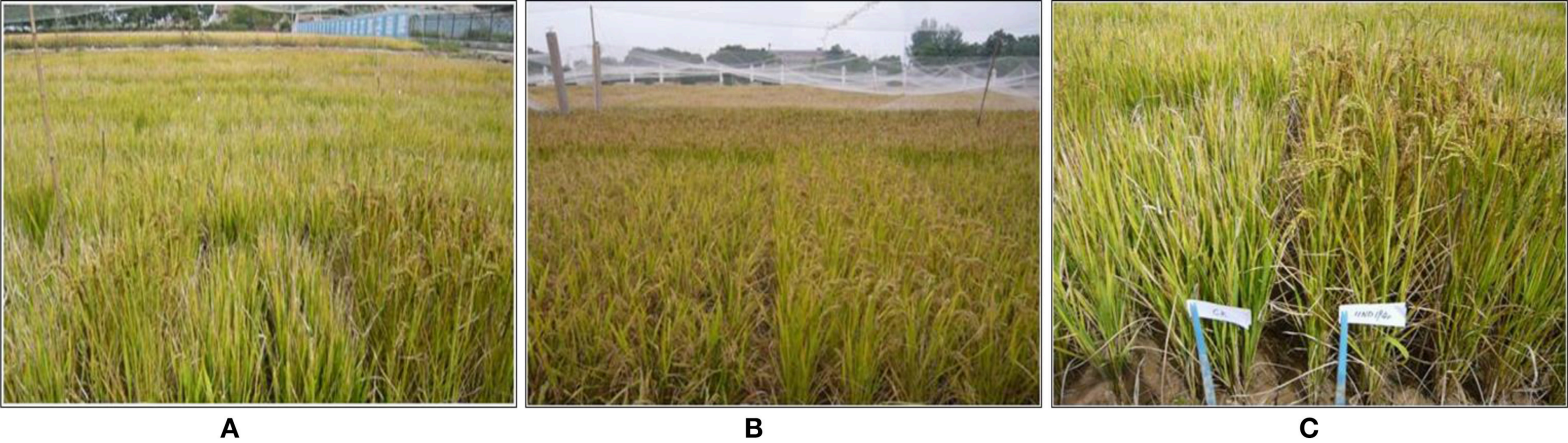
Field screening and performance of drought tolerance under drought stress. **(A)** The field screening of DT at the reproductive stage. **(B)** Performance of the DT ILs under drought stress. **(C)** Comparison of DT IL and JG88 under drought stress (The left side are JG88 and right side are DT ILs).

**Figure 2:**
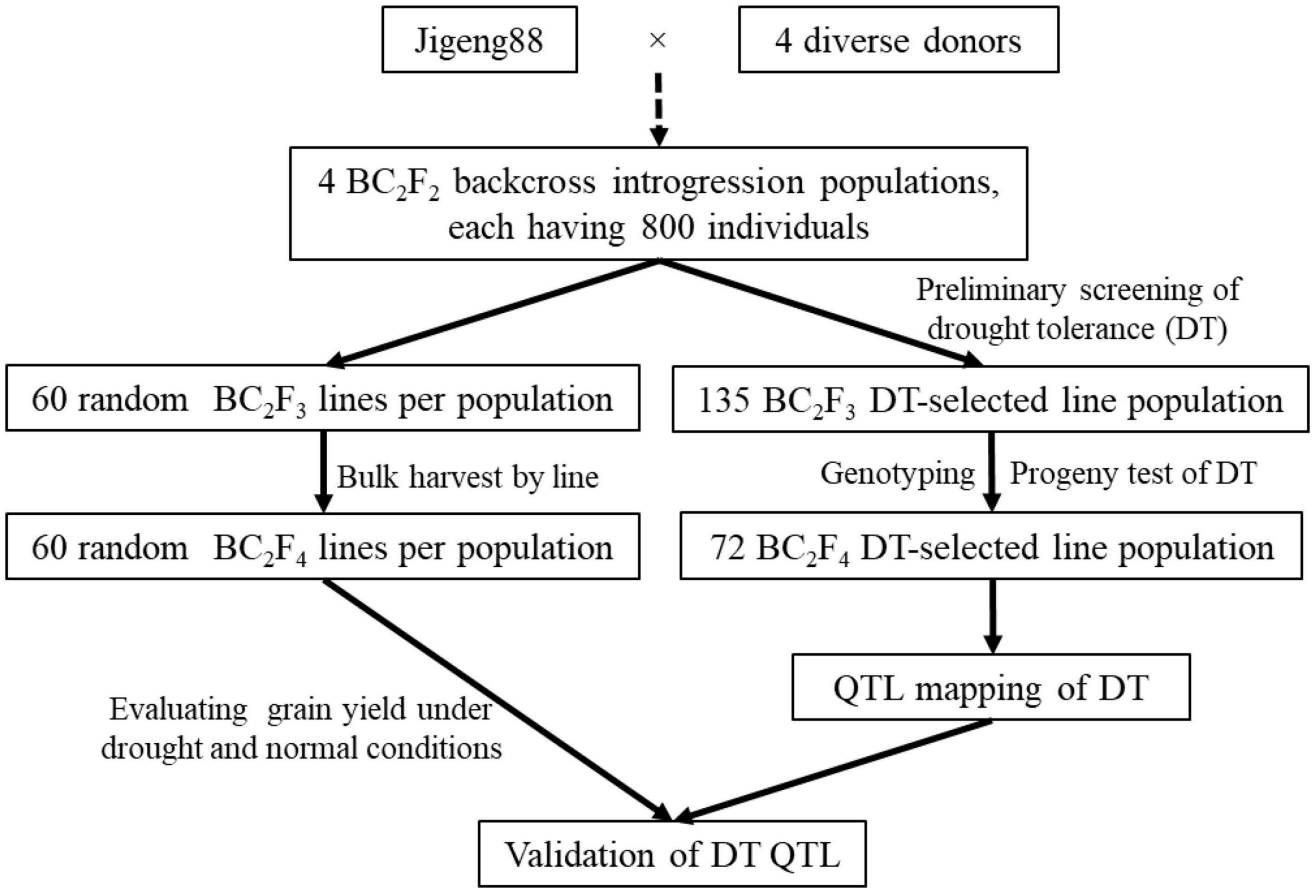
Flowchart of the population development for identification and validation of QTL for drought tolerance in rice.

The screening of the BC_2_F_2_ bulk populations was conducted on the experimental farm of NAAFS. The soil of the test field was a sandy clay. In the initial screen for DT, 800 25-day old seedlings of each BC_2_F_2_ population were transplanted into a big 80-row plot with 10 plants per row and a spacing of 20 × 25 cm between plants and rows flanked by two rows of the RP in the summer of 2008 at the NAAFS. Figure 1A shows the field screening of drought tolerance (DT) at reproductive stage. The field was managed under the normal irrigation until the peak tillering stage 50 days after transplanting. Then, the field was drained. Flush irrigation was applied twice when drought stress became very severe due to large evaporation capacity, at an interval of 10 days (total water applied 1,600 m^3^ ha^−1^), to create severe water stress at the reproductive stage. The resulting soil water content was ~16–19% (v/v) based on constant monitoring using the time domain reflectometry method (TRIME-FM moisture meter; IMKO GmbH, Ettlingen, Germany) at a soil depth of 0–30 cm. No rainfall occurred during this period at the study site. Out of 3,200 plants from the four BC_2_F_2_ populations, total of 135 plants survived under the severe drought stress, and then were harvested individually at the maturing stage. All selected BC_2_F_3_ lines were progeny tested under the similar drought stress (DS) conditions in the summer of 2009, and 72 BC_2_F_3_ lines with significantly higher yield were selected, ranging from 10 lines from the Jigeng88/MR167 population to 24 lines from the Jigeng88/SN265 population (Table 1 and Figures 1B,C). The flowchart shows the population development for identification of QTL in selected population and validation of QTL in random population (Figure 2).

### Phenotypic data collection

In the summers of 2012 and 2013, all selected 72 BC_2_F_4_ lines plus 235 random BC_2_F_4_ lines from the same four populations were evaluated under the drought stress and irrigated conditions in the experimental farms of NAAFS and Chinese Academy of Agricultural Sciences (CAAS) in Beijing (BJ). The soil of the test field was a sandy loam in Beijing. In each experiment, 30-day old seedlings of each ILs were transplanted into a five-row plot with 30 plants in each plot and a spacing of 20 × 25 cm between plants and rows. A completely randomized block design was used with two replications per line. Under the irrigated control condition, water was applied whenever necessary until most lines had reached the grain-filling stage (total water applied 4,800 m^3^ ha^−1^). For drought stress, moderate stress condition was performed to validate the DT lines at the NAAFS. A water sheltered facility was adopted in CAAS for creating drought stress. Normal irrigation was maintained for 1 month after transplanting, then the plots were drained and irrigation was withheld completely till harvest. Thus, all tested lines were subjected to severe drought at the reproductive stage. During the periods of drought stress, water levels of the fields were monitored daily at a soil depth of 0–30 cm based on constant monitoring using the time domain reflectometry method (TRIME-FM moisture meter; IMKO GmbH, Ettlingen, Germany). At maturity, all plants were harvested and measured for grain yield and five plants in each plot were sampled and measured for following yield related traits: heading date (HD), plant height (PH), effective panicle number per plant (PN), thousand-grain weight (GW), filled grain number per panicle (GN), grain weight per plant (GY), and spikelet number per panicle (SN). HD was recorded when the panicle was exerted ~50% of the plants in a plot.

### Genotyping experiments

Genomic DNA was extracted using the CTAB method (Ahmadikhah, 2009). The DNA was isolated from bulk fresh leaf tissues of each BC_2_F_2:3_ IL. More than 600 rice anchor simple sequence repeat (SSR) markers were used to survey the parental lines, resulting in 181, 201, 197, and 38 polymorphic SSR markers, respectively, for the four populations. The markers were used to genotype the selected ILs and random ILs.

### Statistical analysis

Because of the greatly reduced variation among lines within each of the selected IL population, normal statistical methods for identifying QTL in random segregating populations are not appropriate for detecting QTL in selected populations. Therefore, we took a segregation distortion approach to map segregation distortion markers (Cui et al., 2015). In addition, we combined the four selected breeding populations together to perform a joint QTL mapping following the method developed by Cui et al. (2015). However, the four selected populations in this study have different markers. Some markers in some populations were not genotyped. We first developed a consensus map using the multipoint method under the Markov model (Cui et al., 2015). As Cui et al developed, an individual survived from the drought stress with an underlying quantitative trait *y*_*j*_called liability which can be described by the liner model

(1) $$\begin{array}{r} y_{j}=Z_{j}a+\xi_{j} \end{array}$$

where *Z*_*j*_ is the genotype indicator for individual *j*, *a* is the genetic effect of locus, ξ_*j*_ is the residual error follow the normal distribution ξ_*j*_ ~ *N*(0, 1). Assume all the individuals survived are selected based on the *y*_*j*_ > 0 criterion. The probability of surviving is Pr(*y*_*j*_ > 0) = Φ(*Z*_*j*_*a*). Using the Bayes' theorem, the posterior probability of survival for each genotype are $\pi_{j\left(11\right)}=\phi_{11}\Phi \left(a\right)/{\bar{\pi }}_{j}$, $\pi_{j\left(12\right)}=\phi_{12}\Phi \left(0\right)/{\bar{\pi }}_{j}$, $\pi_{j\left(22\right)}=\phi_{22}\Phi \left(-a\right)/{\bar{\pi }}_{j}$, where ${\bar{\pi }}_{j}=\phi_{11}\Phi \left(a\right)+\phi_{12}\Phi \left(0\right)+\phi_{22}\Phi \left(-a\right)$ and ϕ_11_ = 13/16, ϕ_12_ = 2/16, ϕ_22_ = 1/16 are the expected Mendelian frequencies for the three genotypes in BC_2_F_2_. When *a* = 0, the posterior probabilities are equal to the expected Mendelian frequencies and we will not be able to detect segregation distortion. If *a* ≠ 0 the posterior probabilities of genotypes will deviate from the expected Mendelian Segregation ratios. The segregation distortion loci could be identified. For each population, we estimated the effect of each marker and calculated the variance of the estimated effect. Let â_*k*_ be the estimated effect and var(â_*k*_) be the variance of the estimate for a marker under consideration. The Wald test for *H*_0_:*a*_*k*_ = 0 in the *k*th population was obtained using ${â}_{k}^{2}/\mathrm{var}\left({â}_{k}^{}\right)$. The joint test for all populations for *H*_0_:*a*_1_ = *a*_2_ = … = *a*_*p*_ = 0 is

(2) $$\begin{array}{r} \text{Wald}=\overset{p}{\underset{k=1}{\sum }}\frac{{â}_{k}^{2}}{\mathrm{var}\left({â}_{k}\right)} \end{array}$$

where *p* = 4 is the number of populations. When there are multiple populations, the Wald test simply takes the sum of the Wald test of each individual population. In fact, the random model approach was developed by treating each *a*_*k*_ as a normally distributed random variable with a common variance across populations, i.e., $a_{k}\sim N(0,\sigma_{a}^{2})$ for *k* = 1, …, *p*. The shared variance justified the joint mapping (Cui et al., 2015). The critical value for genome-wide significance at the 0.05 level was drawn from 1,000 permuted samples (Churchill and Doerge, 1994).

### Construction of genetic networks underlying drought tolerance

According to the molecular quantitative genetic theory (Zhang et al., 2011; Wang et al., 2015), segregating loci at different levels of signaling pathways contributing to DT in the selected ILs from each BC population were expected to show significant frequency shifts and non-random associations in a strictly hierarchical manner. Here, a functional genetic unit (FGU) is defined either as a single locus of significant excess introgression or an associate group (AG) of *r* (*r* ≥ 2) unlinked but perfectly associated loci of equal introgression in the DT ILs selected from each BC population. Single FGUs of excessive introgression could be detected by the segregation distortion method described above, or by simple *X*^2^ tests for detecting significant deviations of the donor and genotypic frequencies at individual loci across the genome from the expected allelic and genotypic frequencies estimated from all genotyped markers of the random populations. In addition, DT loci involved in epistasis (the same signaling pathways) were expected to show strong non-random associations in response to selection, and thus could be detected as association groups (AGs) each consisting of *r* (*r* ≥ 2) unlinked but perfectly associated loci of equal introgression in DT ILs selected from each BC population. Thus, a multi-locus probability test:

(3) $$\begin{array}{r} P_{\left(AG\right)}={\left(p_{i}\right)}^{rm}\times {\left(1-p_{i}\right)}^{r\left(n-m\right)} \end{array}$$

where *p*_*i*_ is the frequency of the donor introgression in the random ILs from each BC population, *n*is the number of the selected ILs, *m*is the number of ILs that have co-introgression of the donor alleles, and (*n* − *m*) is the number of ILs having no introgression at the *r* unlinked loci in the AG. Here ${\left(p_{i}\right)}^{m}$ is the probability of *m* ILs having co-introgression of the donor alleles and ${\left(1-p_{i}\right)}^{n-m}$ is the probability of (*n* − *m*) ILs having no introgression at *r* unlinked loci. The threshold to claim a significant case was *P* ≤ 0.005 (Zhang et al., 2014). For each AG consisting of *r* (*r* ≥ 2) unlinked but perfectly associated loci, *r* × (*r* − 1)/2 significant pairwise associations would be existed between the *r*loci, which were also confirmed by the linkage disequilibrium (LD) analyses. To reveal the multi-locus structure or putative genetic network underlying DT in the ILs from each BC population, pairwise gametic LD analyses were performed to characterize the relationship between alleles at all DT FGUs detected in the DT ILs from each BC population. The equation of LD statistic

(4) $$\begin{array}{r} {\tilde{D}}_{AB}={\tilde{p}}_{AB}-{\tilde{p}}_{A}{\tilde{p}}_{B} \end{array}$$

where ${\tilde{p}}_{AB}$, $\;{\tilde{p}}_{A}$, and ${\tilde{p}}_{B}$were the frequencies of co-introgression functional AB and functional genotypes at FGUs A and FGUs B, respectively. A multi-locus genetic network including all detected FGUs in the confirmed drought-tolerance ILs was constructed in two steps based on the principle hierarchy: (1) All the FGUs detected in the DT ILs from a single population were divided into major groups according to the LD results. The individual FGUs of different IF within each group were all significantly and positively associated with $\overset{⌢}{D}{s_{AB}}^{\prime }=1.0$, and FGUs in different groups were either independent or negatively associated; (2) Based on the principle of hierarchy, all associated FGUs within each group were connected and formed multiple layers according to their progressively reduced functional genotypes (FG) frequencies and inclusive relationships (Zhang et al., 2011). The underlying assumption for the network construction is that all FGUs in a network are genetically independent (unlinked) from one another, which was true in our cases because all redundant loci due to linkage (recombination frequency ≤ 0.4) associated with each of the FGUs detected in DT ILs of each population were removed.

## Results

### Developing ILs with significantly improved DT and yields

From a total of 3,200 plants in the four BC_2_F_2_ populations, 135 plants survived the drought stress and were selected in the first round screening. The number of selected individuals ranged from 28 in population I to 40 in population II (Table 1). After progeny testing of the 135 DT BC_2_F_3_ lines, 17, 21, 10, and 24 DT ILs from the four populations showed significantly higher GY than the recurrent parent (JG88) under drought stress at the reproductive stage. In the phenotyping experiment, all BC_2_F_4_ IL populations showed significantly improved GY under drought stress when compared to JG88, at Ningxia and/or Beijing (Table 2). The ANOVA results show that there was a statistically significant difference in all traits between the normal and drought stress conditions. The location factor for trait PH, PN, SN, GW, and HD were found to be statistically significant with *P* < 0.01. There was a significantly difference for PH, PN, GW, and HD between the different lines. The G by E interactions were existed in the trait PH and PN (Supplementary Table 1). The mean yield advantage of the IL populations over the RP ranged from 12.7 to 22.5% under the moderate drought stress (JG88 suffered 58.5% yield loss) in Ningxia, and from 89.4 to 178.9% under the severe drought (JG88 suffered 91.0% yield loss) in Beijing, even though there was some residual variation for DT among ILs from each population (Table 2). Clearly, the more severe of drought stress was, the greater the yield advantage over the RP the selected DT ILs had. When the yield related traits of the IL populations were examined, almost all populations had similar mean trait values as JG88 except for population I which had significantly higher trait values for PH, SN, and GN. Under the irrigated conditions, no significant differences were detected for yield and related traits between the selected IL populations and JG88 (Table 2). Nevertheless, we were able to identify five promising ILs which had significant higher yields than JG88 under both drought stress and normal irrigated conditions in both and/or either environments (Table 3).

**Table 2:** Mean performances of the DT introgression lines for grain yield and related traits selected from 4 backcross populations and their recurrent parent (JG88) in Ningxia (2013) and Beijing (2012) under the drought stress and non-stress irrigated conditions.

	**Population**	**Location**	**N**	**GY(g)**	**HD(d)**	**PH(cm)**	**PN**	**GN**	**SN**	**GW(g)**
Drought stress	I	NX	17	8.4 ± 1.8^*^	108.1 ± 6.1	84.5 ± 13.3*	4.7 ± 0.6	121.3 ± 17.0^**^	151.1 ± 20.0^**^	17.8 ± 1.5
	II	NX	21	8.1 ± 1.0^*^	101.6 ± 3.7	67.5 ± 6.7	5.3 ± 0.7^*^	94.0 ± 14.8	126.5 ± 18.9	18.0 ± 1.2
	III	NX	10	8.7 ± 1.5^**^	103.3 ± 4.1	69.8 ± 4.8	5.7 ± 0.8^**^	93.3 ± 16.1	119.4 ± 15.9	19.0 ± 1.7
	IV	NX	24	8.0 ± 1.2^*^	106.4 ± 1.4	74.2 ± 3.3	5.2 ± 0.6	97.4 ± 10.2	111.6 ± 10.6	18.8 ± 1.8
	JG88(RP)	NX		7.1 ± 0.4	105.1 ± 1.9	73.6 ± 2.1	4.7 ± 0.4	87.9 ± 3.3	113.4 ± 15.1	20.3 ± 2.4
	I	BJ	17	4.5 ± 2.1	96.9 ± 8.8^*^	89.1 ± 12.4^*^	2.1 ± 1.2	114.8 ± 46.7	150.4 ± 50.9	14.9 ± 1.9
	II	BJ	21	5.3 ± 1.8	93.3 ± 3.9	79.0 ± 7.1	3.0 ± 1.4	119.0 ± 54.8	170.9 ± 123.4	16.7 ± 1.4
	III	BJ	10	4.6 ± 2.5	91.7 ± 3.3	81.2 ± 5.6	2.2 ± 1.4	130.1 ± 60.7^*^	155.4 ± 62.9	16.1 ± 2.4
	IV	BJ	24	3.6 ± 1.6	98.0 ± 3.0^**^	89.1 ± 5.4^*^	1.9 ± 0.9	111.2 ± 52.8	141.0 ± 66.6	16.1 ± 1.9
	JG88(RP)	BJ		1.9 ± 0.5	92.5 ± 3.2	78.5 ± 6.6	2.2 ± 1.0	97.7 ± 56.2	138.7 ± 60.2	15.7 ± 1.9
Non-stress irrigated conditions	I	NX	17	17.4 ± 3.9	102.5 ± 5.0	95.8 ± 10.7	5.9 ± 1.0	184.1 ± 22.0	204.5 ± 21.7	20.3 ± 1.3
	II	NX	21	18.5 ± 2.7	96.9 ± 2.3	93.3 ± 7.4	6.2 ± 1.2	175.7 ± 26.2	192.5 ± 27.7	21.5 ± 1.4
	III	NX	10	16.9 ± 3.8	99.4 ± 3.9	87.4 ± 6.4	5.9 ± 0.9	157.7 ± 30.5	169.1 ± 31.8	22.3 ± 1.9
	IV	NX	24	18.9 ± 3.1	101.6 ± 1.8	99.4 ± 3.4	6.3 ± 0.9	175.7 ± 20.0	187.3 ± 19.7	21.3 ± 1.2
	JG88(RP)	NX		17.1 ± 1.4	99.0 ± 2.6	91.0 ± 4.8	6.0 ± 0.6	175.1 ± 28.7	186.6 ± 31.8	21.5 ± 2.1
	I	BJ	17	22.6 ± 4.5	93.3 ± 6.8	114.2 ± 14.1^**^	7.3 ± 1.1	217.3 ± 31.9	245.8 ± 29.0	19.1 ± 1.2
	II	BJ	21	20.0 ± 2.4	90.9 ± 5.1	104.4 ± 6.1	6.2 ± 0.8	206.4 ± 37.9	237.2 ± 44.9	19.6 ± 1.2
	III	BJ	10	21.6 ± 6.3	91.5 ± 4.8	100.2 ± 7.1	6.8 ± 1.2	192.6 ± 32.6	208.2 ± 31.5	20.6 ± 1.0
	IV	BJ	24	22.1 ± 2.4	96.2 ± 1.7	109.8 ± 3.6	6.7 ± 0.8	214.4 ± 19.3	226.2 ± 20.3	20.0 ± 1.3
	JG88(RP)	BJ		21.1 ± 4.7	94.9 ± 4.5	106.5 ± 2.1	7.4 ± 1.7	222.0 ± 25.8	241.0 ± 29.3	19.6 ± 2.2

*GY, grain yield; PH, plant height; PN, effective panicle number per plant; GN, filled grain number per panicle; SN, spike number per panicle; GW, thousand grain weight; HD, head date*.

*and ** *indicate the significance levels of P = 0.05 and 0.01, respectively, based on Duncan's multiple comparisons in ANOVA*.

**Table 3:** Five promising JG88 introgression lines with significantly improved yields under both drought stress and irrigated conditions compare with recurrent parent (JG88) in either Ningxia (2013) and/or Beijing (2012) under the drought stress and non-stress irrigated conditions.

**ILs**	**Env**.	**Under drought**							**Irrigated conditions**						
		**GY (g)**	**HD (d)**	**PH(cm)**	**PN**	**FGN**	**SN**	**GW (g)**	**GY (g)**	**HD(d)**	**PH (cm)**	**PN**	**FGN**	**SN**	**GW (g)**
BJC9	BJ	6.9 ± 1.8^*^	97.5 ± 1.5	83.2 ± 1.5	3.3 ± 2.2	97.8 ± 23.8	124.5 ± 10.5	15.5 ± 0.8	21.8 ± 1.9	97.0 ± 0.1	108.3 ± 2.5	6.9 ± 1.5	204.4 ± 20.8	218.7 ± 23.3	20.8 ± 0.2
	NX	10.0 ± 0.7^*^	107.5 ± 1.5	74.6 ± 0.4	4.5 ± 0.1	126.1 ± 0.7^**^	143.0 ± 3.6^*^	19.6 ± 0.1	21.6 ± 3.1	104.1 ± 2.0	91.9 ± 0.3	6.8 ± 1.0	197.3 ± 1.9^*^	220.6 ± 0.6^*^	21.6 ± 0.3
BJC85	BJ	4.2 ± 1.9	89.5 ± 2.5	79.8 ± 1.1	4.7 ± 1.7	120.2 ± 30.1	134.3 ± 31.8	16.2 ± 0.1	24.4 ± 0.6	92.5 ± 0.5	100.8 ± 1.6	7.6 ± 0.8	177.8 ± 3.4	195.1 ± 11.7	20.9 ± 1.3
	NX	8.8 ± 0.3^*^	99.0 ± 0.1^**^	65.3 ± 0.7^*^	5.4 ± 0.1^**^	92.5 ± 6.1	113.7 ± 2.5	19.1 ± 0.5	18.2 ± 2.2	96.1 ± 0.1^*^	92.8 ± 1.0	6.1 ± 1.0	171.1 ± 5.3	180.5 ± 1.7	21.3 ± 0.6
BJC101	BJ	3.1 ± 1.1	97.0 ± 1.3	90.3 ± 0.5^*^	4.3 ± 0.5^*^	103.3 ± 28.3	115.8 ± 36.8	17.5 ± 1.8	21.6 ± 3.0	97.5 ± 1.5	108.9 ± 0.7	6.6 ± 1.0	185.9 ± 2.1	221.9 ± 28.5	21.1 ± 1.3
	NX	10.8 ± 0.7^*^	106.0 ± 0.1	75.5 ± 1.5	6.1 ± 1.1	107.9 ± 5.1	114.0 ± 7.2	20.9 ± 0.1^*^	24.7 ± 6.4	102.0 ± 0.1^*^	101.5 ± 4.3	7.6 ± 1.8	168.1 ± 6.7	174.9 ± 8.1	22.3 ± 0.4
BJC105	BJ	2.5 ± 0.2^*^	98.0 ± 1.7	86.7 ± 0.1	4.1 ± 2.0	166.7 ± 16.5	198.2 ± 7.0	18.4 ± 0.8^*^	25.6 ± 5.9	97.5 ± 0.5	109.1 ± 0.3	8.5 ± 2.3	214.3 ± 3.3	220.1 ± 1.9	19.8 ± 0.7
	NX	8.6 ± 0.5^*^	106.5 ± 0.5	73.0 ± 1.2	4.4 ± 0.1^*^	104.6 ± 6.8	117.2 ± 9.8	19.4 ± 0.1	19.9 ± 2.6	100.5 ± 2.5	99.1 ± 3.3	6.1 ± 0.9	188.2 ± 1.6	198.4 ± 4.4	21.6 ± 0.8
BJC112	BJ	6.3 ± 1.0^*^	98.0 ± 0.5	94.2 ± 2.0^*^	2.5 ± 7.0	113.7 ± 8.7	131.0 ± 47.3	16.1 ± 1.5	22.2 ± 4.5	96.1 ± 1.0	115.3 ± 1.5^*^	5.8 ± 1.0	236.5 ± 11.7	246.4 ± 17.6	20.9 ± 0.3
	NX	8.6 ± 0.7^*^	106.5 ± 0.5	75.9 ± 1.5	5.1 ± 0.5	99.9 ± 3.5	110.4 ± 3.2	19.6 ± 0.2	25.2 ± 7.7	105.5 ± 4.5	105.8 ± 7.8	8.2 ± 2.2	194.1 ± 23.2	207.8 ± 30.0	21.1 ± 1.4
JG88	BJ	1.9 ± 0.5	92.5 ± 3.2	78.5 ± 6.6	2.2 ± 1.0	97.7 ± 56.2	138.7 ± 60.2	15.7 ± 1.9	21.1 ± 4.7	94.9 ± 4.5	106.5 ± 2.1	7.4 ± 1.7	222.1 ± 25.8	241.1 ± 29.3	19.6 ± 2.2
	NX	7.1 ± 0.4	105.0 ± 1.9	73.6 ± 2.1	4.7 ± 0.4	87.9 ± 3.3	113.4 ± 15.1	20.3 ± 2.4	17.1 ± 1.4	99.1 ± 2.6	91.1 ± 4.8	6.0 ± 0.6	175.1 ± 28.7	186.6 ± 31.8	21.5 ± 2.1

*The trait abbreviations are the same as Table 2*.

*and** *indicate the significance levels of P = 0.05 and 0.01, respectively, based on Duncan's multiple comparisons in ANOVA*.

### Detection and validation of DT QTL in the selected and random IL populations

Using a critical value of Wald test of 16.97 drawn from 1,000 permutated samples, 13 QTLs on seven rice chromosomes were detected in 72 ILs selected from the four BC populations (Table 4). These included 7 QTL (*QDT1.3, QDT2.4, QDT2.9, QDT7.1, QDT7.2, QDT7.4*, and *QDT11.5*) in the 17 ILs from population JG88/IR66897B(I), 7 QTL (*QDT1.4, QDT6.3, QDT2.9, QDT6.3, QDT6.5*, and *QDT10.3*) from 21 ILs from population JG88/MR77(II), 3 QTL (*QDT2.9, QDT6.3*, and *QDT6.5*) from 10 ILs from population JG88/MR167 (III), and 4 QTL (*QDT1.3, QDT2.4, QDT6.3*, and *QDT6.5*) from the 24 ILs of JG88/SN265(IV). Of these, 2 QTL (*QDT2.9* and *QDT6.3*) each was detected in three of the populations, 4 QTL (*QDT1.3, QDT1.4, QDT6.5*, and *QDT7.1*) each was detected in two of the populations, and the 7 remaining QTL each was detected in a single population. Two QTL, *QDT1.4* near marker RM449 on chromosome 1 and *QDT2.9* near MR266 on chromosome 2 were identified with the highest Wald values of 65.8 and 41.54.

**Table 4:** Thirteen QTL associated with drought tolerance identified by the segregation distortion mapping method in DT introgression lines selected from four backcross populations.

**QTL**	**Marker**	**Position (cM)^a^**	**Wald value^b^**	**Population^c^**	**QTL and drought-responsive genes**
*QDT1.3*	RM572	66.4	22.15	I, II	*rfw1b* (Li et al., 2005a)
*QDT1.4*	RM449	78.4	65.8	II, IV	*brt1d* (Li et al., 2005a)
*QDT2.4*	RM424	66	17.5	I	*qDTY2.1* (Dixit et al., 2012); *qLRS-2* (You et al., 2006)
*QDT2.8*	RM425	166	32.72	II	
*QDT2.9*	RM266	192.2	41.54	I, II, III	*OsPIP1;3* (Lian et al., 2004; Liu et al., 2007)
*QDT6.3*	RM276	40.3	37.87	II, III, IV	
*QDT6.5*	RM541	75.5	27.82	II, IV	*qgy6.1* (Palanog et al., 2014)
*QDT7.1*	RM427	1.1	20.74	I, IV	*OsCIPK23* (Yang et al., 2008)
*QDT7.2*	RM125	24.8	25.12	I	*OsNAC3/ONAC067* (Ooka et al., 2003)
*QDT7.4*	RM542	34.7	30.42	I	*rn7a* (Li et al., 2005a)
*QDT8.3*	RM339	72.2	22.05	III	*QGy8* (Xu et al., 2005); *QPn8,QTgw8, QSf8* (Wang et al., 2012)
*QDT10.3*	RM311	25.2	19.82	II	*trdw10.1* (Nguyen et al., 2004)
*QDT11.5*	RM229	77.8	21.75	I	

a *cM means centimorgan, a unit of genetic distance*.

b *Wald value = 16.97 and 20.35 at P = 0.05 and 0.01, respectively*.

c *The designations of the populations are the same as Table 1*.

In order to validate the detected DT QTL, the four random (unselected) populations each with ~60 BC_2_F_4_ lines from the same four crosses were evaluated under drought stress in the replicated trials. Based on the genotypic differences in mean yields under drought at each of the detected QTL in the random BC_2_F_4_ lines from each population, 11 of the 13 detected QTL could be validated in the random populations evaluated in the Ningxia experiment and 8 of the DT QTL could be validated in the random populations in the Beijing experiment (Table 5). In all cases, significantly increased GY under drought stress in both Ningxia and Beijing were associated with the donor homozygote genotype, indicating that the donor alleles at all these detected QTL were associated with DT. The average yield advantage under drought from the donor homozygote at individual QTL ranged from 4.1% for *QDT8.3* to 45.9% for *QDT10.3*.

**Table 5:** Phenotype validation of DT QTLs by comparing mean grain yields of QTL genotypes of the four random populations in Ningxia (2013) and Beijing (2012) under the drought condition.

**QTL**	**Marker**	**Population**	**Mean grain yield (g/plant) in Ningxia**			**Mean grain yield (g/plant) in Beijing**			**Yield improvement^a^ (%)**
			**Donor homozygote**	**JG88 homozygote**	**Jg88**	**Donor homozygote**	**JG88 homozygote**	**Jg88**	
*QDT1.4*	RM449	IV	9.22	8.47	7.71	7.54	6.60	6.89	14.5
*QDT2.4*	RM424	I	10.30	8.8	7.38				39.6
*QDT2.8*	RM425	II	10.25	8.46	7.63	8.38	6.87	6.52	31.4
*QDT2.9*	RM266	I	10.23	9.05	7.38	6.39	5.91	5.41	28.4
		II	9.92	8.68	8.82				12.5
*QDT6.3*	RM276	II	9.83	9.04	8.82	8.23	7.00	6.52	18.8
*QDT6.5*	RM541	II	10.10	8.58	7.63	8.60	6.81	6.42	24.2
		IV				8.20	6.70	6.89	19.0
*QDT7.1*	RM427	I	11.56	8.72	8.82	6.86	6.10	5.41	28.9
		IV	9.03	8.31	7.71				17.1
*QDT7.4*	RM542	I	9.52	9.10	8.82				8.0
*QDT8.3*	RM339	III	9.18	8.26	8.82				4.1
*QDT10.3*	RM311	II	11.13	9.05	7.63				45.9
*QDT11.5*	RM229	I	10.00	9.10	7.38	7.04	6.07	5.41	32.8

a *Yield improvement (%) = (mean GY of ILs carrying the donor allele – mean GY of JG88)/mean GY of JG88*.

### Putative genetic networks (multi-locus structures) underlying DT

**Figure 3:**
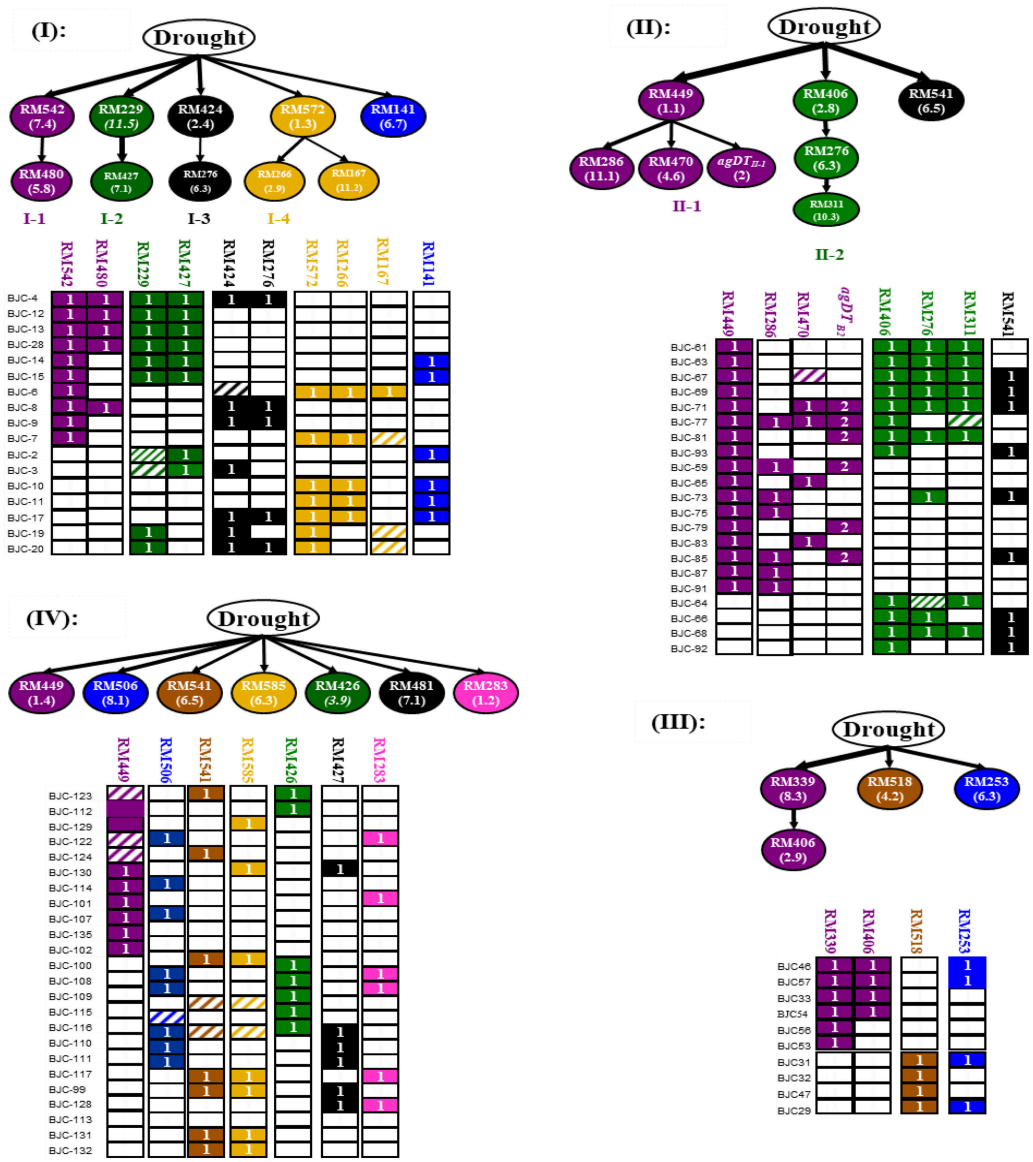
Putative genetic networks (multi-locus structures) underlying drought tolerance (DT) of rice detected in JG88 backcross introgression lines (BILs) from four populations. In the corresponding graphical genotypes of each network, the unfilled, fully colored, and patched cells represent the recipient homozygote, donor homozygote, and heterozygote genotypes. The numbers in the cells of each FGU are the number of loci included in the FGUs. The loci (markers) included in each of the detected association groups (AGs) are shown in Table 6. Solid arrow lines connected two FGUs in each branch of a network represent putative functional relationships with those of high introgression as putative regulators in the upstream and those of low introgression in the downstream, and the thickness of an arrow line was proportional to the introgression frequency of the downstream FGU in Table 6.

Table 6 shows 29 FGUs (28 single loci and 1 association group or AG) for DT detected by χ^2^ tests (single loci) and multi-locus linkage disequilibrium analyses in 72 drought-tolerant introgression lines (ILs) selected from the four populations. These included 10 FGUs detected in 17 ILs of JG88/IR66897B (I), 9 loci in eight FGUs in 21 ILs of JG88/MR77 (II), 4 FGUs in 10 ILs of JG88/MR167 (III), and 7 FGUs in 24 ILs of JG88/SN265(IV), respectively (Table 6 and Figure 3). The average introgression frequency (IF) of the donor alleles at the 30 DT loci ranged from 0.147 to 0.81.

**Table 6:** Genomic information for 29 functional genetic units (FGUs) (28 single loci and 1 association groups or AGs) for drought tolerance (DT) detected by χ^2^ tests (single loci) and multi-locus linkage disequilibrium analyses in 72 drought-tolerant introgression lines (ILs) selected from four populations.

**Donor**	**Code**	**AG^a^**	**Branch**	**Marker**	**Bin^b^**	**Position (cM)^c^**	**B^d^**	**H**	**IF**	***P*-value**
IR66897B	I		I-1	RM542	**7.4**	34.7	10	0	0.588	1.40E-11
IR66897B	I		I-1	RM480	5.8	130.6	5	0	0.294	1.49E-02
IR66897B	I		I-2	RM229	**11.5**	77.8	8	2	0.529	2.66E-10
IR66897B	I		I-2	RM427	**7.1**	1.1	8	0	0.471	4.70E-07
IR66897B	I		I-3	RM424	**2.4**	66	7	1	0.441	6.66E-06
IR66897B	I		I-3	RM276	**6.3**	40.3	4	0	0.235	1.67E-02
IR66897B	I		I-4	RM572	**1.3**	61.2	7	0	0.412	3.38E-05
IR66897B	I		I-4	RM266	**2.9**	192.2	5	0	0.294	1.67E-02
IR66897B	I		I-4	RM167	11.2	37.5	1	3	0.147	6.35E-08
IR66897B	I			RM141	6.7	143.7	6	0	0.353	9.50E-04
MR77	II		II-1	RM449	**1.1**	78.4	17	0	0.81	9.56E-29
MR77	II	*agDT_*II*−1_*	II-1	RM336	7.6	61	6	0	0.286	8.36E-03
MR77	II	*agDT_*II*−1_*	II-1	RM331	8.3	69	6	0	0.286	8.36E-03
MR77	II		II-1	RM286	**11.1**	0	7	0	0.333	6.32E-04
MR77	II		II-1	RM470	4.6	115.5	4	1	0.214	1.14E-01
MR77	II		II-2	RM406	2.8	186.4	12	0	0.571	3.03E-13
MR77	II		II-2	RM276	**6.3**	40.3	9	1	0.452	9.35E-06
MR77	II		II-2	RM311	**10.3**	25.2	9	1	0.452	2.14E-07
MR77	II			RM541	**6.5**	75.5	9	0	0.429	6.53E-07
MR167	III		III-1	RM339	**8.3**	72.2	6	0	0.6	2.04E-07
MR167	III			RM406	2.9	186.4	4	0	0.4	3.39E-03
MR167	III			RM518	4.2	25.5	4	0	0.4	3.39E-03
MR167	III			RM253	6.3	37	4	0	0.4	3.39E-03
SN265	IV			RM449	**1.4**	78.4	8	3	0.396	4.95E-08
SN265	IV			RM506	8.1	0	8	1	0.354	1.81E-04
SN265	IV			RM541	**6.5**	75.5	7	2	0.333	5.77E-05
SN265	IV			RM585	6.3	25.1	7	2	0.333	5.77E-05
SN265	IV			RM426	3.9	157.3	7	0	0.292	4.42E-03
SN265	IV			RM481	**7.1**	3.2	6	0	0.25	3.28E-02
SN265	IV			RM283	1.2	31.4	6	0	0.25	3.28E-02

a *AGs are defined as a group unlinked but perfectly associated loci of equal introgression in the selected DT ILs from each BC population, detected by multi-locus probability tests. P-value is the probabilities for the null hypothesis that the genotypic frequencies fit the Mendelian segregation based on single locus X^2^ tests*.

b *Bold ones were DT QTLs detected by the segregation distortion approach in Table 2*.

c *cM means centimorgan, a unit of genetic distance*.

d *B, H, and IF are the frequencies of the donor homozygote, heterozygote, and donor introgression frequency in the selected DT ILs from each population*.

Figure 3 shows the four putative genetic networks or multi-locus structures each containing all DT FGUs identified in the selected DT ILs from populations I–IV. Genetic network I contains 10 FGUs (10 loci) in five largely independent branches detected in the 17 DT ILs from population Jigeng88/IR66897B. Branch I-1 consisted of two unlinked but perfectly associated loci, with RM542 (bin 7.4) of high introgression placed in the upstream (IF = 0.588) and RM480 (bin 5.8) of lower introgression (IF = 0.294) in the downstream. Similarly, branches I-2, I-3, and I-4 each also consisted of two unlinked but perfectly associated loci with a locus of high introgression placed in the upstream and a locus of low introgression placed in the downstream. Branch I-5 contains a single locus near RM141 (bin 6.7). Genetic network II consisted of eight FGUs (9 loci) with two major branches plus two independent loci detected in the 21 DT ILs from population Jigeng88/MR77. Branch II-1 had RM449 (Bin1.1) of very high introgression (IF = 0.810) in the upstream as the putative regulator connected with three sub-branches, RM286 (bin11.1) (IF = 0.333), RM470 (bin 4.6) (IF = 0.214), and *agDT*_*II*−1_ consisting of two unlinked but perfectly associated loci at RM336 (Bin7.6) and RM331 (Bin8.3) of low introgression (IF = 0.286) in the downstream. Branch II-2 consisted of 3 unlinked but highly associated FGUs with RM406 of high introgression placed at the top as the putative regulator and 2 FGUs (RM276 in Bin6.3 and RM311 in Bin10.3) of lower introgression in the downstream. The independent FGUs included RM541 at bin 6.5 of high introgression (IF = 0.429) which were detected as DT QTL using the segregation distortion method (Table 3). Genetic network III detected in the 10 ILs of JG88/MR167 consisted of 4 FGUs (4 loci) with a single branch plus two independent loci (MR518 in bin4.2 and MR253 in bin6.3). Branch III-1 containing 2 unlinked but highly associated loci with MR339 (bin8.3) in the upstream and MR406 (bin2.9) in the downstream. The genetic network of JG88/SN265 contained only 7 independent loci (FGUs). This was not surprising since both JG88 and SN265 are closely related *Geng* varieties from Northeast China.

## Discussion

In this study, we have shown that most selected ILs from the four BC populations had significantly improved GY under drought stress without yield penalty under the normal irrigated conditions (Table 2). This result plus the development of five promising ILs that had significantly higher yields under both drought stress and normal irrigated conditions indicated that backcross breeding was effective for improving DT of high yielding *Geng* varieties. We noted that the improved yield performances of ILs under drought stress were associated primarily with increased PN and GN, but not with increased TGW. This is consistent with the effect of natural selection which tends to act on the number instead of size of rice grains (Ashikari et al., 2005; Lu et al., 2013). It should be pointed out that none of the four donors in our BC breeding has good DT, but they apparently all have genes contributing to DT, indicating the presence of rich “hidden” genetic diversity in the primary gene pool of rice for DT, as reported previously (Lafitte et al., 2006; He et al., 2010; Wang et al., 2013a,b; Ali et al., 2017). This is true for tolerances to other abiotic stresses such as salinity, submergence, high and low temperatures, etc. (Ali et al., 2006; Cheng et al., 2011; Zhang et al., 2014) as well as for almost all complex traits in rice (Li and Rutger, 2000; Zhang, 2012).

Rice responses to drought and other abiotic stresses are known to be controlled by complex gene networks consisting of many signaling pathways (Wang et al., 2011, 2015). However, it remains a great challenge to link results from the classic QTL mapping with those of molecular and transcriptomic analyses. Resembling the previously reported genetic networks underlying rice tolerances to cold and submergence (Zhang et al., 2014; Wang et al., 2015), the genetic networks underlying DT detected in this study each consisted of multiple independent branches, each might represent a signaling pathway involved in rice DT based on the following pieces of evidence. First, all DT QTL of large effects (high introgression) detected in this study (Table 4) appeared to be real ones as they were all validated in the random BC populations of small population sizes that are known to be less powerful in detecting QTL. Secondly, seven of the major DT QTL (*QDT1.3, QDT1.4, QDT2.4, QDT6.5, QDT7.4, QDT8.3*, and *QDT11.5*) had high introgression and were placed as putative regulators in the upstream positions of the genetic networks (Figure 3), as predicted by the theory (Zhang et al., 2011). Two other putative regulators at bins 1.3 (branch I-4) and 2.8 (branch II-2) were also in the approximate vicinity to *QDT1.4* and *QDT2.9*, suggesting they were likely due to linkage. Thirdly, according to the QTLs/genes located in the region within 200 kb are the same QTL/gene, we found that most of the major QTL identified in this study were mapped to approximately the same locations as previously reported QTL or important regulatory genes for DT (Table 4). For example, *QDT7.2* (near RM125) was mapped to the genomic location harboring *ONAC067/OsNAC3*, a member of plant-specific NAC family that is known to regulate plant responses to drought, cold and high salinity (Kikuchi et al., 2003; Takasaki et al., 2010). This region also harbor two previously reported DT QTL in rice detected in a set of recombinant inbred lines developed from the cross between Zhenshan 97 (*Xian*) and a upland rice cultivar IRAT109 (*tropical Geng*; Yue et al., 2005, 2008). Similarly, *QDT2.9* was mapped together with *OsPIP1-3*, a gene that showed increased transcription in response to drought and probably played an important role in drought avoidance in rice (Lian et al., 2004). The *QDT7.1* was mapped in the region with a cloned gene *OsCIPK23* which is a multi-stress induced gene mediates a signaling pathway commonly shared by both pollination and drought stress. The *QDTY2.4* was mapped to the same location as most a previously reported DT QTL, *qDTY2.1*, and *qLRS-2* (Dixit et al., 2012). The *QDT1.3* and *QDT1.3* located on chromosome 1 were mapped in the adjacent region harboring *rfw1b* and *brt1d* (Li et al., 2005a). The *QDT8.3* was mapped in the same region of *QGy8* for grain yield, *QPn8* for panicle number, *QTgw8* for thousand grain weight and *QSf8* for seed fertility (Wang et al., 2012) under drought stresses. The *QDT10.3* was mapped to the same region as the *trdw10.1* was reported by Nguyen et al. (2004). The *QDT2.8, QDT6.3*, and *QDT11.5* are probably new DT QTLs which have been validated in random population of this study. All these results strongly suggested that most main-effect QTL detected in this study were most likely regulatory genes that play important roles in regulating rice responses to DT and probably other abiotic stresses. We noted that 8 additional downstream FGUs by our non-random association analyses were undetectable by the segregation distortion method (SDM). This was due partially to the fact that SDM considers only allelic frequency shifts but not on the deviation of genotypic frequency shifts from direction selection, and primarily to its inability to detect epistasis (Cui et al., 2015).

Finally, the DT ILs and the genetic information regarding the DT QTL and the network (Supplementary Table 2) they carry provide useful materials and information for further improving rice DT and yield by designed QTL pyramiding (Zhang et al., 2014; Ali et al., 2017). As we noted in Table 2, there were considerable residual variation among individual ILs for GY and related traits, and so were for different QTL from different donors in their genetic compositions (Figure 1). According to our experiences, it is hoped that better and promising progeny that combine higher levels of DT and grain yield can be readily achieved using this breeding strategy with relatively short period of time and limited breeding effort (Ali et al., 2017).

## Author contributions

YC analyzed the data and wrote the manuscript; WZ and XL were in charge of the field experiment management; SX developed the statistical model; JX designed and performed the experiment; ZL conceived the study and were in charge of the direction and planning.

### Conflict of interest statement

The authors declare that the research was conducted in the absence of any commercial or financial relationships that could be construed as a potential conflict of interest.
