# Supplementary material for: Simultaneous Improvement and Genetic Dissection of Drought Tolerance Using Selected Breeding Populations of Rice
Source: Front Plant Sci. 2018 Mar 15;9:320. doi: 10.3389/fpls.2018.00320 (PMC5862857; doi:10.3389/fpls.2018.00320)
Supplement: Supplementary file 1 [file Table1.DOCX]

**Supplementary Table 1** The ANOVA results of the selected population

| Item | Df | GY | | PH | | EP | | PN | | SN | | GW | | HD | |
| --- | --- | --- | --- | --- | --- | --- | --- | --- | --- | --- | --- | --- | --- | --- | --- |
|  |  | F value | P value | F value | P value | F value | P value | F value | P value | F value | P value | F value | P value | F value | P value |
| Gene | 71 | 1.0 | 0.464 | 15.1 | <0.01^**^ | 0.6 | 0.995 | 1.9 | <0.01^**^ | 1.6 | 0.002 | 3.6 | <0.01^**^ | 14.3 | <0.01^**^ |
| Location | 1 | 0 | 0.857 | 513.0 | <0.01^**^ | 74.3 | 0.000 | 88.0 | <0.01^**^ | 94.2 | <0.01^**^ | 208.2 | <0.01^**^ | 888.1 | <0.01^**^ |
| Environment | 1 | 1223.8 | <0.01^**^ | 1709.7 | <0.01^**^ | 326.8 | <0.01** | 668.0 | <0.01^**^ | 350.1 | <0.01^**^ | 565.1 | <0.01^**^ | 144.8 | <0.01^**^ |
| G*E | 71 | 0.9 | 0.724 | 1.4 | 0.024^*^ | 0.5 | 0.999 | 1.4 | 0.009^**^ | 1.3 | 0.076 | 0.9 | 0.648 | 0.7 | 0.955 |

Note: * and ** indicate the significance levels of P = 0.05 and 0.01, respectively, based on Duncan’s multiple comparisons in ANOVA

**Supplementary Table 2** The information on SSR markers linked with DT QTL and DT network detected in selected populations

| Marker | Accession number | GenBank ID | Clone name | Chr. | Position (cM) | Repeat type | Size | Forward primer | Reverse primer |
| --- | --- | --- | --- | --- | --- | --- | --- | --- | --- |
| RM283 | AF344109 | 14210004 | CT788 | 1 | 31.4 | (GA)18 | 147-155 | gtctacatgtacccttgttggg | cggcatgagagtctgtgatg |
| RM572 | AP001072 | 6721501 | P0025D05 | 1 | 66.4 | (TC)14 | 147-181 | cggttaatgtcatctgattgg | ttcgagatccaagactgacc |
| RM449 | AQ291851 | 3953045 | OSJNBa0040K15 | 1 | 78.4 | (AG)12 | 116-138 | ttgggaggtgttgataaggc | accaccagcgtctctctctc |
| RM424 | AQ258181 | 3782663 | OSJNBa0019H06 | 2 | 66 | (CAT)9 | 239-290 | tttgtggctcaccagttgag | tggcgcattcatgtcatc |
| RM425 | AQ259183 | 3783665 | OSJNBa0022C06 | 2 | 166 | (CGG)9 | 120-126 | ccaacgaagattcgaagctc | cagcaccatgaagtcgcc |
| RM406 | AQ259248 | 3783730 | OSJNBa0022I06 | 2 | 186.4 | (GA)17 | 126-146 | gagggagaaaggtggacatg | tgtgctccttgggaagaaag |
| RM266 | AF344093 | 14209988 | CT483 | 2 | 192.2 | (GA)19 | 121-137 | tagtttaaccaagactctc | ggttgaacccaaatctgca |
| RM426 | AQ259184 | 3783666 | OSJNBa0022C08 | 3 | 157.3 | (CA)10 | 150-224 | atgagatgagttcaaggccc | aactctgtacctccatcgcc |
| RM518 | AQ690034 | 5331202 | OSJNBa0081M01 | 4 | 25.5 | (TC)15 | 153-187 | ctcttcactcactcaccatgg | atccatctggagcaagcaac |
| RM470 | AQ330314 | 4122164 | OSJNBa0046N10 | 4 | 115.5 | (CTT)14 | 83-122 | tcctcatcggcttcttcttc | agaacccgttctacgtcacg |
| RM480 | AQ364445 | 4214100 | OSJNBa0061C13 | 5 | 130.6 | (AC)30 | 199-225 | gctcaagcattctgcagttg | gcgcttctgcttattggaag |
| RM585 | AB026295 | 5295936 | P0681F10 | 6 | 25.1 | (TC)45 | 171-259 | cagtcttgctccgtttgttg | ctgtgactgacttggtcatagg |
| RM253 | n.a. | n.a. | CT506 | 6 | 37 | (GA)25 | 125-143 | tccttcaagagtgcaaaacc | gcattgtcatgtcgaagcc |
| RM276 | AF344102 | 14209997 | CT715 | 6 | 40.3 | (AG)8A3(GA)33 | 85-153 | ctcaacgttgacacctcgtg | tcctccatcgagcagtatca |
| RM541 | AQ862610 | 6213067 | OSJNBb0019O09 | 6 | 75.5 | (TC)16 | 156-192 | tataaccgacctcagtgccc | ccttactcccatgccatgag |
| RM141 | D48997 | 702706 | n.a. | 6 | 143.7 | (CT)12 | 126-145 | caccaccaccaccacgcctctc | tcttggagaggaggaggcgcgg |
| RM427 | AQ271555 | 3824870 | OSJNBa0026M19 | 7 | 1.1 | (TG)11 | 173-185 | tcactagctctgccctgacc | tgatgagagttggttgcgag |
| RM481 | AQ364700 | 4214355 | OSJNBa0061O24 | 7 | 3.2 | (CAA)12 | 136-172 | tagctagccgattgaatggc | ctccacctcctatgttgttg |
| RM125 | D39885 | 569036 | n.a. | 7 | 24.8 | (GCT)8 | 124-136 | atcagcagccatggcagcgacc | aggggatcatgtgccgaaggcc |
| RM542 | n.a. | n.a. | n.a. | 7 | 34.7 | (CT)22 | 91-113 | tgaatcaagcccctcactac | ctgcaacgagtaaggcagag |
| RM336 | AF344160 | 14210055 | CTT53 | 7 | 61 | (CTT)18 | 148-193 | cttacagagaaacggcatcg | gctggtttgtttcaggttcg |
| RM506 | AQ576484 | 4976969 | OSJNBa0089C06 | 8 | 0 | (CT)13 | 115-123 | cgagctaacttccgttctgg | gctacttgggtagctgaccg |
| RM339 | AF344163 | 14210058 | CTT85 | 8 | 72.2 | (CTT)8CCT(CTT)5 | 142-160 | gtaatcgatgctgtgggaag | gagtcatgtgatagccgatatg |
| RM311 | AF344136 | 14210031 | GT147 | 10 | 25.2 | (GT)3(GTAT)8(GT)5 | 164-186 | tggtagtataggtactaaacat | tcctatacacatacaaacatac |
| RM286 | AF344112 | 14210007 | CT806 | 11 | 0 | (GA)16 | 99-128 | ggcttcatctttggcgac | ccggattcacgagataaactc |
| RM167 | n.a. | n.a. | n.a. | 11 | 37.5 | (GA)16 | 127-159 | gatccagcgtgaggaacacgt | agtccgaccacaaggtgcgttgtc |
| RM229 | AF344049 | 14209944 | CT224 | 11 | 77.8 | (TC)11(CT)5C3(CT)5 | 106-131 | cactcacacgaacgactgac | cgcaggttcttgtgaaatgt |

Note: n.a means not available
